# P-2228. Utility of Microbial Cell-Free DNA Next-Generation Sequencing for Evaluation of Vascular Graft Infection

**DOI:** 10.1093/ofid/ofae631.2382

**Published:** 2025-01-29

**Authors:** Melissa Kerkelis, Vaishnavi Ramesh, Nischal Ranganath, Omar M Abu Saleh, Hussam Tabaja

**Affiliations:** Mayo Clinic, Rochester, Minnesota; Mayo Clinic, Rochester, Minnesota; Mayo Clinic, Rochester, Minnesota; Mayo Clinic Rochester, Rochester, Minnesota; Mayo Clinic, Rochester, Minnesota

## Abstract

**Background:**

Vascular graft infection (VGI) is a rare but serious condition. Pathogen identification can be difficult given the deep-seated nature. Microbial cell-free DNA next-generation sequencing (mcfDNA NGS) is an emerging tool in non-invasive microbiologic workup. One commercially available mcfDNA is the Karius Test (KT). There is a paucity of data regarding utility and impact of mcfDNA NGS testing in the evaluation of VGI.

**Figure:**
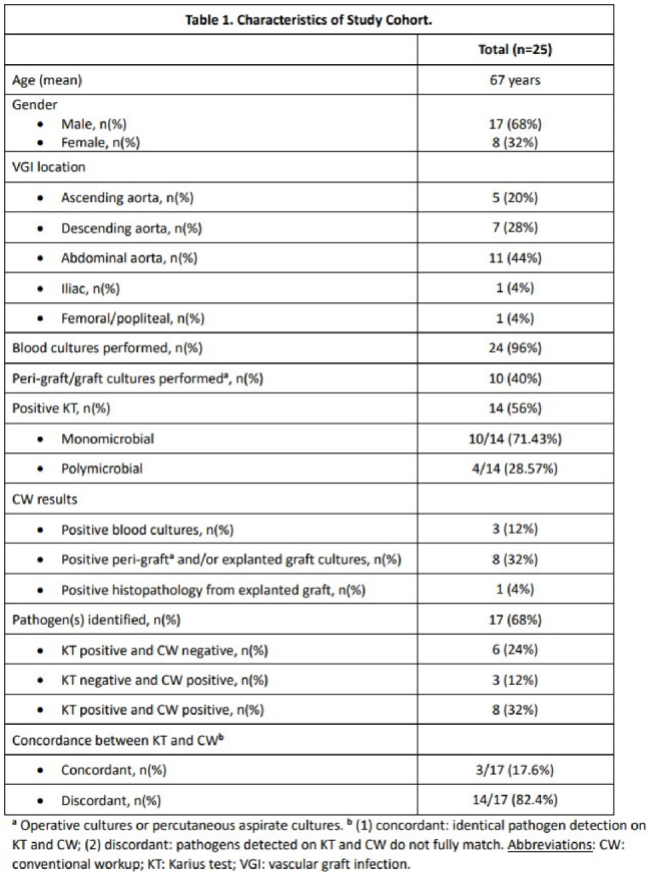

**Methods:**

We retrospectively evaluated adult patients who underwent KT for evaluation of VGI at our institution. Only patients who met the Management of Aortic Graft Infection Collaboration (MAGIC) classification for suspected or confirmed VGI were included. We investigated the concordance between KT and conventional microbiologic workup. Conventional workup (CW) included pathogen identification through blood cultures, peri-graft/graft cultures, or histopathology. Concordance was defined by identical pathogen identification on KT and CW. We examined the influence of the KT result on clinical management.

**Figure 1.**
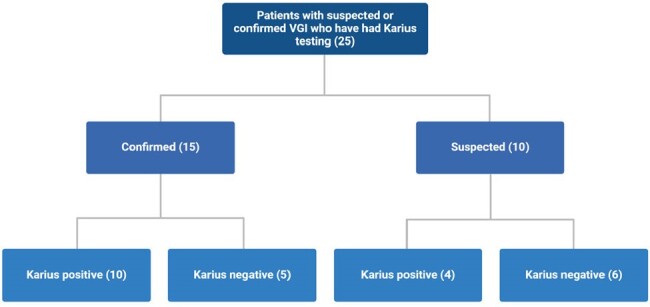
KT Results for Confirmed and Suspected VGI.

**Results:**

We identified 25 patients who met criteria for either suspected or confirmed VGI per the MAGIC criteria. Microbiology was undefined in 8 patients who had negative KT and negative CW. A total of 17 patients had positive workup with 6 having positive KT and negative CW, 3 having negative KT and positive CW, and 8 having both positive KT and positive CW (Table 1). KT was less likely to be positive for patients who met criteria for suspected infection (4/10; 40%) as opposed to confirmed infection (10/15; 66.67%), albeit non-significant (OR 0.33, p=0.188) (Figure 1). The KT led to antimicrobial de-escalation in 27.3% of patients with positive CW and 35.7% of patients with negative CW (Figure 2). Full concordance of KT and CW was noted in 3 patients (Table 2).

**Figure 2.**
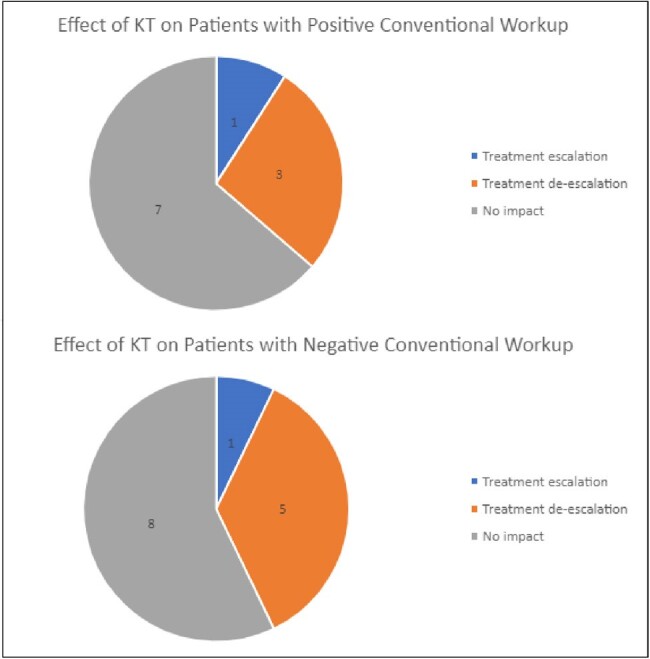
Influence of KT on VGI cases with positive and negative conventional work-up.

Treatment escalation was defined as the broadening of antimicrobial coverage due to KT results. Treatment de-escalation was defined as narrowing/discontinuation of antimicrobial coverage due to KT results.

**Conclusion:**

We found that the positivity rate for KT was 56%. KT impacted management in 40% of cases. Patients with confirmed VGI had a higher likelihood of having a positive KT. KT was the only positive microbiological definitive test in one case. Further studies are needed to explore the utility and impact of KT in the workup of VGI.

**Figure:**
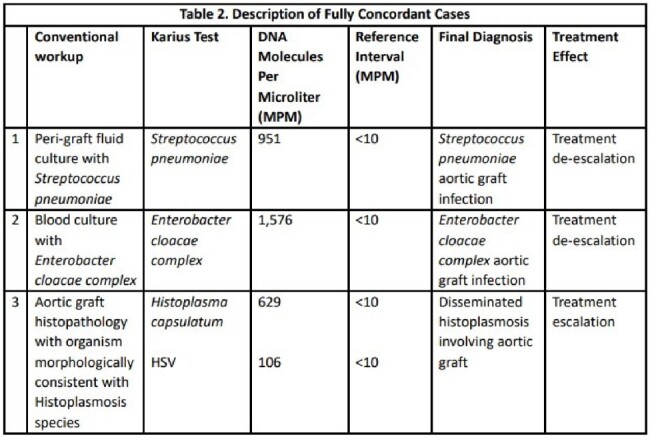

**Disclosures:**

All Authors: No reported disclosures

